# Formulations of Curcumin Nanoparticles for Brain Diseases

**DOI:** 10.3390/biom9020056

**Published:** 2019-02-08

**Authors:** María L. Del Prado-Audelo, Isaac H. Caballero-Florán, Jorge A. Meza-Toledo, Néstor Mendoza-Muñoz, Maykel González-Torres, Benjamín Florán, Hernán Cortés, Gerardo Leyva-Gómez

**Affiliations:** 1 Laboratorio de Posgrado en Tecnología Farmacéutica, FES-Cuautitlán, Universidad Nacional Autónoma de México, Cuautitlán Izcalli 54740, Mexico; ml.delprado@iim.unam.mx; 2 Departamento de Fisiología, Biofísica & Neurociencias, Centro de Investigación y de Estudios Avanzados del Instituto Politécnico Nacional, Ciudad de México 07360, Mexico; hiram.qfohead@gmail.com (I.H.C.-F.); bfloran@fisio.cinvestav.mx (B.F.); 3 Departamento de Farmacia, Facultad de Química, Universidad Nacional Autónoma de México, Ciudad Universitaria, Circuito Exterior S/N, Del. Coyoacán, C.P. Ciudad de México 04510, Mexico; jamtoledo90@outlook.com; 4 Escuela de Ciencias de la Salud, Universidad del Valle de México, Campus Coyoacán, Ciudad de México, 04910, Mexico; 5 Facultad de Ciencias Químicas, Universidad de Colima, C.P. Colima 28400, México; nmendoza0@ucol.cmx; 6 CONACyT-Laboratorio de Biotecnología, Instituto Nacional de Rehabilitación Luis Guillermo Ibarra Ibarra, Ciudad de México 14389, Mexico; mikegcu@gmail.com; 7 Instituto Tecnológico y de Estudios Superiores de Monterrey, Campus Ciudad de México 14380, Mexico; 8 Laboratorio de Medicina Genómica, Departamento de Genética, Instituto Nacional de Rehabilitación Luis Guillermo Ibarra Ibarra, Ciudad de México 14389, Mexico

**Keywords:** curcumin, nanoparticles, inflammation, protein aggregation, brain diseases, Alzheimer’s disease, Parkinson’s disease

## Abstract

Curcumin is a polyphenol that is obtained from *Curcuma longa* and used in various areas, such as food and textiles. Curcumin has important anti-inflammatory and antioxidant properties that allow it to be applied as treatment for several emerging pathologies. Remarkably, there are an elevated number of publications deriving from the terms “curcumin” and “curcumin brain diseases”, which highlights the increasing impact of this polyphenol and the high number of study groups investigating their therapeutic actions. However, its lack of solubility in aqueous media, as well as its poor bioavailability in biological systems, represent limiting factors for its successful application. In this review article, the analysis of its chemical composition and the pivotal mechanisms for brain applications are addressed in a global manner. Furthermore, we emphasize the use of nanoparticles with curcumin and the benefits that have been reached as an example of the extensive advances in this area of health.

## 1. Introduction

Curcumin is an active natural polyphenol component of *Curcuma longa*. Due to its chemical structure, this molecule could be applied in several different fields, such as food, textile, and the pharmaceutical industry. It has been shown that curcumin possess anti-inflammatory and antioxidant properties [1,2]. It also presents a spread spectrum of molecular targets such as transcription factors and their receptors, growth factors, cytokines, genes, and adhesion molecules. For example, curcumin could inhibit the cell signaling pathway of nuclear factor kappa B (NF-κB), which is an important cellular target of cancer cells [3,4,5]. Additionally, the blockade of NF-κB triggers the reduction in the expression of different NF-κB-regulated products, such as tumor necrosis factor alpha (TNF-α), interleukin 8 (IL-8), and cyclooxygenase 2 (COX-2), which play key roles in the inflammation process [6]. Furthermore, it has been recently demonstrated that curcumin may inhibit protein aggregation, such as amyloid-β (Aβ) protein, which is related to several neurological pathologies, such as Alzheimer’s disease (AD) [7].

For these reasons, in recent years, there has been an increasing interest in curcumin-based treatments as managements for many disorders, such as brain diseases. However, its poor bioavailability, low solubility in aqueous media, instability in body fluids, and elevated degradation rate have limited the therapeutic applications of this drug. Different strategies, such as the use of nanotechnology, have emerged to tackle these problems. In general, nanoparticles-based drug delivery systems present important advantages, such as a long lifetime circulation, ability to improve the drug’s aqueous solubility as well the bioavailability, and the capacity to overcome physiological barriers [8,9,10].

Many authors have demonstrated that curcumin-loaded nanoparticles comprise a very effective and attractive treatment for several diseases. Therefore, the main objective of this work is to present an extensive review of the properties of curcumin, the nanotechnology-based curcumin delivery systems, and its potential application for the treatment of brain diseases, particularly AD, Parkinson’s disease (PD), and cancer.

## 2. Curcumin Chemical Information

**Figure 1:**
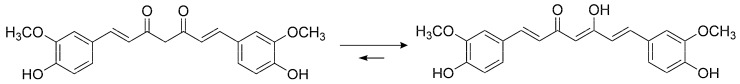
Chemical structure of curcumin and keto–enol tautomerism.

Chemically, curcumin is a naturally polyphenol denominated (1E,6E)-1,7-bis(4-hydroxy-3-methoxyphenyl)-1,6-heptadiene-3,5-dione) (Figure 1), which is isolated from the rhizomes of *C. longa*. From a structural point of view, there are three chemical entities in the molecule: two aromatic ring systems containing *o*-methoxy phenolic groups linked by a seven-carbon spacer consisting of an α,β-unsaturated β-diketone moiety [11]. Therefore, the diketo group exhibits keto–enol tautomerism, meaning that curcumin can exist in equilibrium between the keto and the enol tautomer. However, nuclear magnetic resonance (NMR) studies carried out on a variety of solvents concluded that the enol form of curcumin is essentially the only form of this molecule in solution [12]. The relevance of the tautomerism was explored by Yanagizawa et al. [13]; these authors suggested that curcumin and its analogues exist predominantly in the enol form during binding to Aβ fibrils/aggregates, in turn suggesting that the enolization of curcumin derivatives is crucial for binding to Aβ aggregates in the treatment of AD. In this respect, some physicochemical properties are described below.

### 2.1. Thermal Analysis of Curcumin

**Figure 2:**
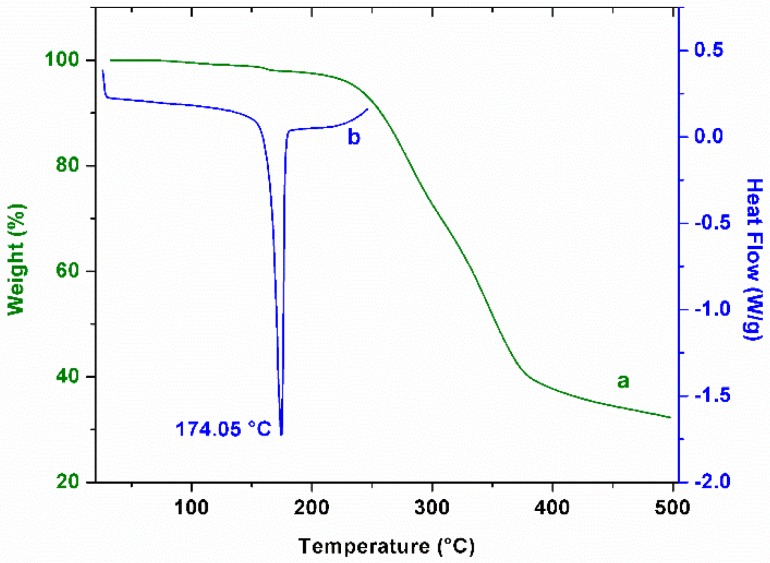
Thermal analysis of curcumin. Thermogravimetric analysis ((a), green line) and differential scanning calorimetry ((b), blue line). Melting point of curcumin is indicated at 174.05 °C.

Thermogravimetric analysis is a common complementary tool to describe this molecule. Therefore, we performed an evaluation of the thermal degradation of curcumin at a heating rate of 10 °C/min and under a nitrogen atmosphere. As can be observed in Figure 2a, the initial temperature of the mass loss is approximately 193 °C. This behavior results in the decomposition of the turmeric powder; below this temperature, weight loss in the curcumin follows a gradual decrease related to the loss of moisture. In a complementary way, a differential scanning calorimetry thermogram (Figure 2b) showed a melting temperature for curcumin of 174.05 °C, which is in agreement with data reported previously [14].

### 2.2. Ultraviolet-Visible Spectrophotometric Analysis of Curcumin

**Figure 3:**
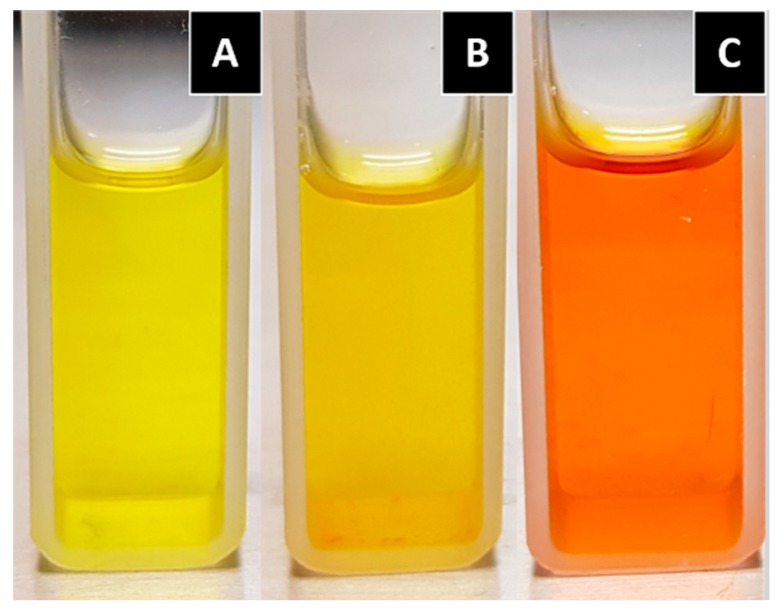
Curcumin dissolved in different mediums. (**A**) Curcumin in an acidic solution (pH 3.5); and (**B**) curcumin in a neutral solution (pH 7.4); both with the addition of 1% Tween 80 in order to increase solubility. (**C**) Curcumin in a basic solution (pH 12).

The chemical reactivity and solubility of curcumin depends on the medium pH in which it is dissolved; that is, under acidic conditions, curcumin exhibits moderate solubility, and the solution maintains a yellow color (Figure 3A), whereas at a neutral pH, curcumin is not fully soluble, as can be observed in Figure 3B. On the other hand, within the basic pH range, curcumin is more water-soluble than in the neutral form, and the color of the solution changes to red (Figure 3C). The color change under alkaline conditions could be an effect deriving from the deprotonation. It is known that the photophysical and photochemical properties of curcumin are related to the solvents polarity because of the keto–enol structure of curcumin that involves intramolecular proton transfer [15]. Kharat M. et al. [16] mentioned the formation of condensation yellow products (such as feruloymethane) as a potential reason for this color increment under an alkaline environment.

**Figure 4:**
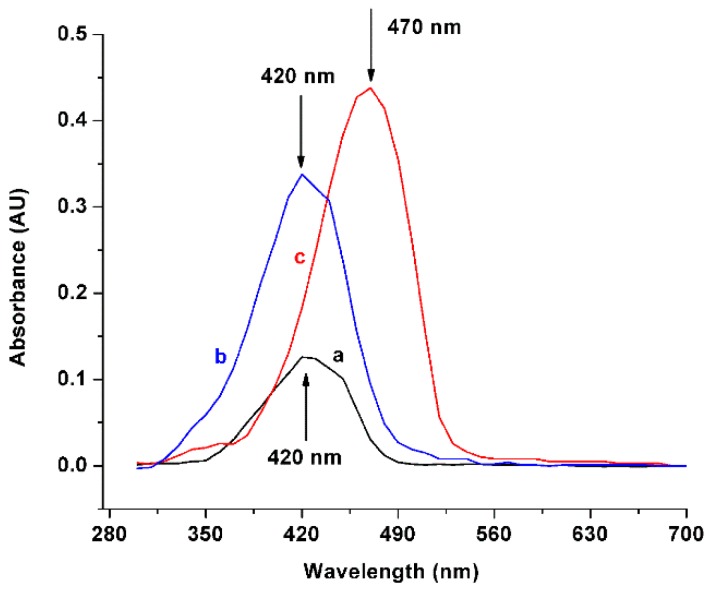
Ultraviolet-Visible spectrophotometric scanning of curcumin. (a) Absorption in methanol, maximum peak of absorption found at 420 nm; (b) Absorption in neutral medium, maximum peak of absorption found at 420 nm; and (c) Absorption in basic medium, maximum peak of absorption found at 470 nm.

In order to corroborate the solubility of curcumin, we tested the drug incorporated in solvents with different pH levels (maintaining a concentration of eight mg/mL), through ultraviolet (UV)-vis spectrophotometry. Figure 4 depicts that at a neutral pH (lines a and b), the maximum absorption peak of curcumin was found at 420 nm, which is in agreement with the literature [17], while the maximum absorption in alkaline pH was found at 470 nm (Figure 4, line c). This result is in agreement with the reported by Priyadarsini [11], who reported that the maximum absorption peak of the fully deprotonated curcumin is found at 467 nm under alkaline conditions (>pH 10).

### 2.3. Fourier Transform Infrared Spectroscopy of Curcumin

**Figure 5:**
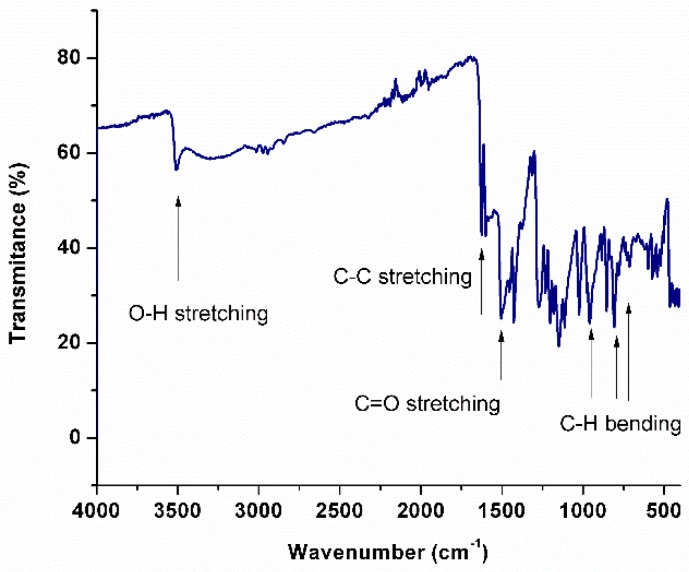
Fourier transform infrared spectroscopy of curcumin. Characteristic bands of the molecule are indicated with arrows.

Infrared spectroscopy is commonly employed to study the molecules’ chemical structure; a curcumin spectrum is shown in Figure 5. As depicted, the characteristic band of the O—H bond stretching appears at 3506 cm^−1^. The infrared (IR) band at 2915 cm^−1^ and its doublet at 2847 cm^−1^ are due to the asymmetric and symmetric stretching vibrations of the C–H_2_ group. For C–C stretching, a peak at 1624 cm^−1^ is found. The C=O stretching vibration of the carboxylic groups (methyl esters and triglycerides) can be attributed to the strong band at 1510 cm^−1^ [18]. In plane C–OH bending vibration can be assigned to the IR bands at 1375 cm^−1^. Curcumin shows a peak at around 1270 cm^−1^, which corresponds to the C—O stretching frequency of the ether group in curcumin. The peaks at 729 cm^−1^, 806 cm^−1^, and 955 cm^−1^ indicate the bending vibrations of the —CH bond of the alkene group [19].

### 2.4. Solubility

Curcumin has poor solubility in water (an estimated of 3.21 mg/L at 25 °C); however, it is soluble in ethanol, dimethyl sulfoxide (DMSO), methanol, acetonitrile, chloroform, and ethyl acetate [11,20]. The theoretical Hansen solubility parameters (HSP), which were calculated on the basis of the group contribution method, are δ_d_ = 17.46, δ_p_ = 3.66, δ_h_ = 13.84, and δ_total_ = 22.46 for the enol form of curcumin [21]. The first pKa_1_ = 7.5–8.5 corresponds to the deprotonation of the enolic proton group, while pKa_2_ = 8.5–10.4 and pKa_3_ = 9.5–10.7 are for the phenolic protons, indistinctly [11]. The log octanol–water partition coefficient (log Kow) is 3.29 (estimated), conferring hydrophobic characteristics on the molecule [20].

Curcumin is unstable in aqueous and alcoholic solution, and it is more stable in acidic pH (1.2–6) than in alkaline pH (Figure 3); the degradation products found under hydrolytic conditions are: trans-6-(4′-hydroxy-3′-methoxyphenyl)-2,4-dioxo-5-hexenal, ferulic acid, ferulic aldehyde, feruloylmethane, and vanillin [22,23]. However, new evidence suggests that the major degradation product is bicyclopentadione, which is the result of the autoxidation of curcumin [24], and is formed by the oxygenation and double cyclization of the heptadienedione chain connecting the two methoxyphenol rings of curcumin [25]. Curcumin is also sensitive to light. It has been shown to decompose when it is exposed to UV/visible light, both in solution and in the solid state [26].

## 3. Biological Activity

**Figure 6:**
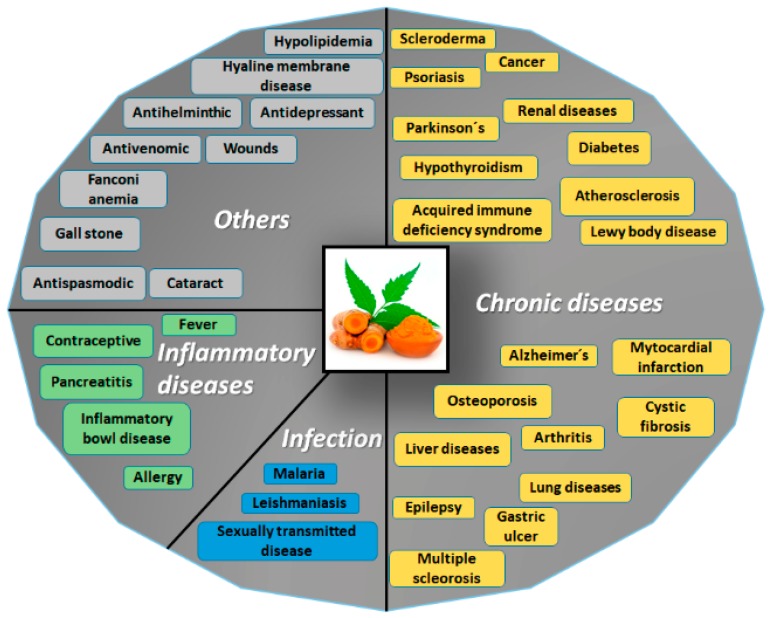
Potential applications of curcumin. Due to the structure of curcumin, this molecule could be applied as treatment for a wide range of disorders, such as chronic diseases, inflammatory disorders, infections of diverse etiology, and other conditions. Adapted with permission from [26]. 2007, Springer Nature.

Curcumin has a broad spectrum of biological activities. For example, it has been reported as possessing antioxidant, anti-AD, anticarcinogenic, antimutagenic, and anti-inflammatory properties (Figure 6).

With respect to antioxidant activity, evidence has shown that curcumin can directly scavenge several free radicals as the result of its two phenolic sites. Likewise, curcumin has been effective against the generation of reactive oxygen species (ROS) and reactive nitrogen species (RNS) in the cellular environment. Curcumin also reduces low-density lipoprotein (LDL), and inhibits the oxidation of proteins and DNA. At the enzymatic level, curcumin inhibits lipoxygenase/cyclooxygenase and xanthine dehydrogenase/oxidase, which are two enzymes related to the generation of ROS, and upregulates superoxide dismutase and glutathione peroxidase, which are two first-line enzymes of defense against oxygen-free radicals [22].

In AD, curcumin protects against Aβ-induced oxidative stress, prevents the formation and extension of Aβ fibrils, destabilizes Aβ fibrils, inhibits acetylcholinesterase, decreases neuroinflammation, and sequesters transition metals [22]. A variety of structure–activity studies have proven that the three moieties in the chemical structure of curcumin play different roles in its interaction with the Aβ peptide: one of the hydroxyl substitutions in the aromatic end group is necessary for inhibition, while the other one of the hydroxyl substitutions is required for activity. Finally, the diketo chain contributes to the flexibility and correct length between aromatic rings [27]. Curcumin has been extensively evaluated and possesses potential antioxidant and anti-inflammatory activity in AD. The most important mechanism of the anti-inflammatory action of curcumin is based on the inhibition of NF-kB, which leads to the decreased formation of cytochemokines and Aβ fibrils. Other molecular targets inhibited by curcumin are inducible nitric oxide synthase (iNOs), c-Jun N-terminal kinase (JNK) activation, and activating protein-1 (AP-1) [28].

**Figure 7:**
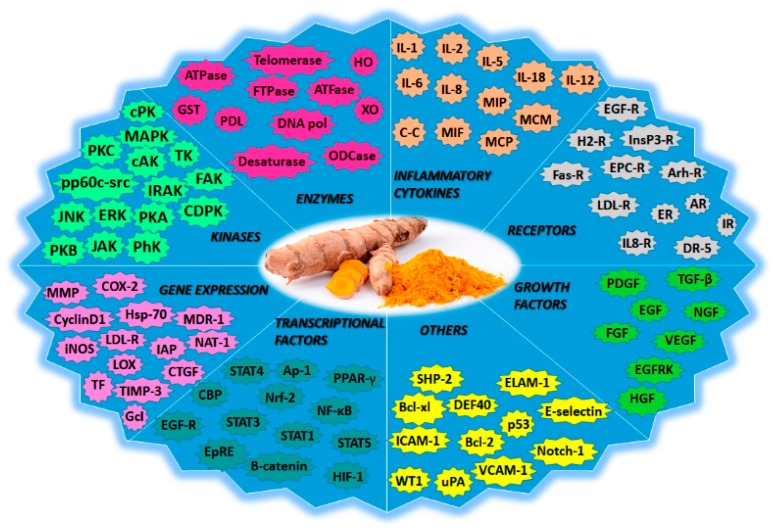
Curcumin is a pleiotropic agent with multiple molecular targets. This molecule could modify the expression of genes, inflammatory cytokines, transcriptional and growth factors, enzymes, and receptors, among others. Adapted with permission from [26]. 2007, Springer Nature.

In anticancer therapy, curcumin inhibits oxidative stress, reduces lipid peroxidation and DNA single-strand breakage, inhibits the COX-1 and COX-2 enzymes, suppresses NF-kB activation, and possesses antiproliferative effects. Moreover, it induces apoptosis by targeting mitochondria, and affects tumor protein p53 (p53)-related signaling [22]. The specific molecular targets for curcumin that are therapeutically important in cancer-signaling pathways include cyclin-dependent kinases (CDKs), p53, Ras, phosphoinositide 3-kinase (PI3K), Protein kinase B (Akt), Wnt/β-catenin, and mammalian target of rapamycin (mTOR) [29]. During angiogenesis, curcumin can inhibit and/or downregulate the expression of various pro-angiogenic growth factors such as the vascular endothelial growth factor (VEGF), fibroblast growth factor (FGF), and the endothelial growth factor (EGF) [30]. An overview of the molecular targets of curcumin is represented in Figure 7.

### 3.1. Effect of Curcumin on Aggregation Protein

Protein aggregation is the process by which misfolded proteins assume a conformation that cause their polymerization into aggregates and organized fibrils. The adequate aggregation of protein is a precise progression that requires extensive guidance from an excellent control network, which comprises approximately 800 proteins in humans. Many neurodegenerative diseases are associated with inappropriate protein aggregation [31]. These neuronal diseases include disorders in which the aggregates may accumulate in the nucleus, such as for example in polyglutamine expansion diseases (such as spinocerebellar ataxias and Huntington’s disease (HD)), which are pathologies that are characterized by inclusions in cytoplasm (for example, α-synuclein in PD), disorders in which the aggregates are found outside of the cell (prion diseases), or both intracellularly and extracellularly (such as Aβ in AD) [31].

The effect of curcumin on prion disease has been studied by several authors. Hafner-Bratkovič et al. [32] reported that the binding of curcumin to the α-intermediate could block conformational change into the β-structure, and that the binding of curcumin to prion fibrils could prevent further growth, thus, the formation of new seeds. Similarly, Caughey et al. [33] concluded in their work that curcumin inhibits prion protein resistance (PrP-Res) accumulation in neuroblastoma cells infected with the scrapie agent. In addition, these authors reported the partial inhibition of the conversion of PrP into PrP-res.

Additionally, Pandey et al. [34] analyzed the curcumin effect both in vitro and in cell culture models of α-synuclein aggregation. The authors concluded that curcumin induces the inhibition of α-synuclein aggregation in a dose-dependent manner. Also, their results suggested that curcumin increased α-synuclein solubility in cells containing aggregates.

The oligomerization of α-synuclein aggregates is structurally similar to the Aβ-protein aggregates of AD. Therefore, curcumin has been investigated as a potential AD treatment. Brahmkhatri et al. [35] reported that curcumin-loaded gold nanoparticles inhibited Aβ aggregation, and that these were capable of dissolving aggregates. Likewise, Mithu et al. [36] reported that curcumin disorganizes Aβ fibrils; the disruption of Aβ-fibrils was achieved by means of structural changes in the salt bridge region and near the C terminus. A more detailed report on the inhibition of Aβ aggregation revealed that, besides curcumin inhibiting fibril formation in vitro, it also inhibited the formation of Aβ oligomers and their toxicity in vivo [37].

It has been reported that amyloid formation could be limited by mechanisms such as metal chelation [38], and reducing the induction of the β-secretase enzyme (BACE1) by proinflammatory cytokines [39]. It has been suggested that BACE1 has a main role in the initiation of the formation of Aβ [40]; therefore, it is an attractive drug target for AD. The sequential proteolytic cleavage of the Aβ precursor protein (APP), which is a type I transmembrane protein, produced the formation of Aβ.

Zhang et al. [7] studied the interaction between curcumin and Aβ. These authors proposed the modulation of APP levels in the secretory pathway as the cellular mechanism by which curcumin reduces Aβ levels. In addition, they reported that the use of curcumin considerably increased the retention of immature APP in the endoplasmic reticulum. Furthermore, the authors suggested that APP endocytosis could be attenuated by treatment with curcumin.

In order to identify the chemical features that are most important for preventing Aβ accumulation, Reinke et al. [27] examined the effect of three features on the inhibition of amyloid aggregation: the presence of aromatic groups at both extremes of the molecule, the substitution pattern of these aromatics, and the distance and flexibility of the linker section. They demonstrated that the presence of just one single aromatic group did not decrease the protein aggregation; thus, the curcumin efficiency as an aggregation inhibitor could be related to its two phenyl groups. Also, their results suggested that the substitution of these aromatics groups is important for activity, since these are capable of taking part in hydrogen bonding. In addition, the authors reported the approximate distance between the docking sites, which are found between eight and 16 Å from each other; this is similar to the distance between the terminal aromatic regions of curcumin.

### 3.2. Effect of Curcumin in Neuroinflammation

In recent years, interest in the identification and application of natural compounds that limit neuroinflammation has been growing. The term neuroinflammation has been used to describe several different pathological events, from modifications in the morphology of glial cells to fully fledged tissue invasion and destruction by leukocytes. Neuroinflammation plays a key role in the progression of neurodegenerative diseases and in the invasion of central nervous system (CNS) parenchyma by leukocytes, it is one of its main characteristics, as well as a severe loss of the blood–brain barrier (BBB) integrity [6].

**Figure 8:**
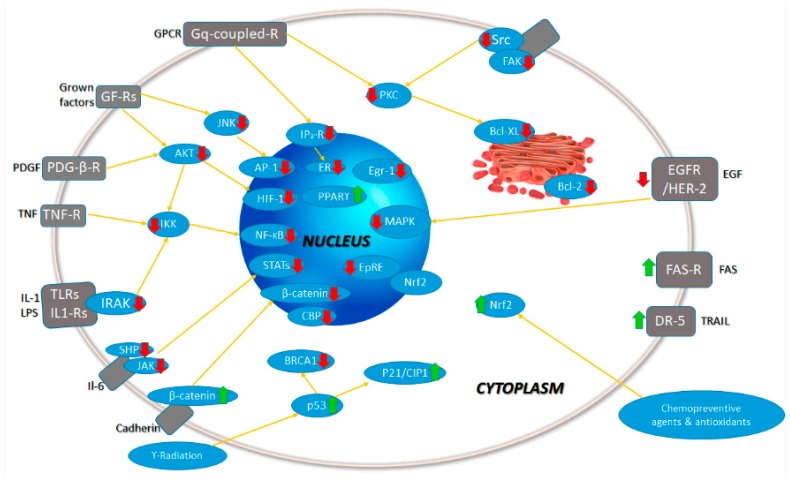
Signaling pathways modulated by curcumin. Up and green arrows indicate the intermediaries upregulated by curcumin; meanwhile, down and red arrows indicate the intermediaries downregulated by curcumin. Adapted with permission from [26]. 2007, Springer Nature.

Due to the latter, lymphocytes and myeloid cells express cytokines in the tissue, increasing the inflammatory cascade. Interleukins such as IL-1β, IL-6, and IL-23, and cytokines such as TNF, interferon gamma (IFNγ), and granulocyte/macrophage colony-stimulating factor (GM-CSF), chemokines (such as CCL2, CCL5, and CXCL1), secondary messengers (nitric oxide and prostaglandins), and ROS are also mediators for the neuroinflammatory response [41]. The excessive production of these inflammatory mediators could cause neuronal damage and death. It has been demonstrated that curcumin reduces the expression of several inflammatory cytokines, including IL-1α, IL-1β, IL-6, TNF, IFNγ, and many others (Figure 8) [42,43].

Some authors have reported that curcumin suppresses the expression of IL-1β [44,45]. This mechanism suggests that curcumin inhibits the activation of the Nod-like receptor protein 3 (NLRP3) inflammasome, which is the most characterized inflammasome, and an important innate immune sensor. The NLRP3 inflammasome is activated by an extensive variety of signals of pathogenic, endogenous, and environmental origin. Some authors found that curcumin suppressed inflammation via a strong inhibition of NLRP3-dependent caspase-1 activation and IL-1 β secretion [44].

In the same manner, Devi et al. [46] reported that curcumin could exert a direct effect on constitutive signal transducer and activator of transcription 3 (STAT3) phosphorylation. These authors also mentioned that curcumin is a potent inhibitor of IL-6 expression in stromal cells. They suggested that this inhibition was related to the IκB kinase (IKK)/NF-κB pathway. Likewise, NF-κB is a ubiquitous transcription factor; its activation is linked with the promotion or inhibition of apoptosis, depending on the cell type and condition. Likewise, the inflammatory response induced by TNF-α is strongly linked to the activation of NF-kB.

On the other hand, TNF-α is a very important inflammatory mediator; thus, its reduction is a therapeutic target in several inflammatory diseases [47]. For the interaction of the TNF-α and curcumin, Wang et al. [4] found inhibition in the expression of this factor through downregulating the expression of NF-κB. These authors reported that curcumin reduced the transcription and secretion of TNF-α, and also IL-6, which is induced by palmitate, and induces the nuclear translocation of NF-κB in a concentration-dependent manner [48,49].

Similarly, the suppression of CCL2 function may decrease the attraction of cells of the immune system to the sites of inflammation, which could result in a slowdown of the advancement of inflammation [50]. Zhang et al. [51] investigated the inhibitory effect of curcumin on lipopolysaccharide (LPS)-induced chemokine CCL production. First, these authors induced the upregulation of CCL2 mRNA and protein in C6 cells, utilizing one µg/mL of LPS. After this, they probed three doses of curcumin as treatments (2.5 µM, 10 µM, and 25 µM), and found that the expression of CCL2 mRNA decreased with these concentrations in a dose-dependent manner. Moreover, their results suggested that in astrocytoma cells, curcumin induces the downregulation of CCL2 expression through the JNK pathway. However, it appears that CCL2 could be inhibited by curcumin through other pathways besides the JNK pathway. Herman et al. [52] suggested that curcumin downregulates CCL2 activity via the inhibition of protein kinase C (PKC) and matrix metalloproteinases.

On the other hand, IFN-γ exerts important effects on epithelial integrity, promotes barrier dysfunction, and increases epithelial permeability through numerous mechanisms. The loss of integrity in BBB is involved in neuronal diseases. For example, Midura-Kiela et al. [53] studied the interaction of IFN-γ and curcumin in epithelial cells. They concluded that curcumin induces the inhibition of the signaling of IFN-γ. Furthermore, curcumin also inhibits the COX-2 pathway. COX-2 is overexpressed in malignant tissues. Accordingly, its inhibition could be a key to cancer and tumor treatments.

### 3.3. Effect of Curcumin on Oxidative Stress

Although oxygen plays an important role in energy production processes in cells (ATP), this molecule could be transformed into very toxic forms in the cells, which are denominated ROS [54]. Antioxidant systems are complex and act in concert to decrease the ROS load. A process known as oxidative stress occurs if an imbalance between ROS and antioxidant systems takes place in favor of oxidants. Oxidative stress has been related to mechanisms leading to neuronal cell injury in various neuronal diseases.

Antioxidants are compounds that can prevent biomolecules from undergoing oxidative damage through free radical-mediated reactions [55]. The majority of the antioxidants present a phenolic group or a β-diketone group in their structure [56].

To explain the protective properties of phenolic groups in antioxidants, two different mechanisms have been proposed. In the first mechanism (Equation (1)), the free radical becomes a radical by hydrogen atom transfer from the antioxidant (ArOH). This antioxidant activity is related to phenolic O–H bond dissociation enthalpy (BDE); thus, if the O–H bond attraction is weak, free radical inactivation will be easier.

In the second mechanism, the free radical receives an electron from the antioxidant. The free radical becomes a radical cation, which could be reacting with another antioxidant molecule (Equations (2) and (3)). In terms of this mechanism, the important parameter to analyze for antioxidant activity is the ionization potential (IP). With a low IP, electron abstraction will be easier. In addition to BDE and IP, proton dissociation enthalpy, proton affinity (PA), and electron transfer enthalpy are important factors for evaluating antioxidant activity [55,57].

(1) $$ArOH+R^{*}\;\to \;ArO^{*}+RH,$$

(2) $$ArOH+R^{*}\;\to \;ArOH^{*}+R^{-},$$

(3) $$ArOH+R^{-}\;\to \;ArO^{*}+RH.$$

**Figure 9:**
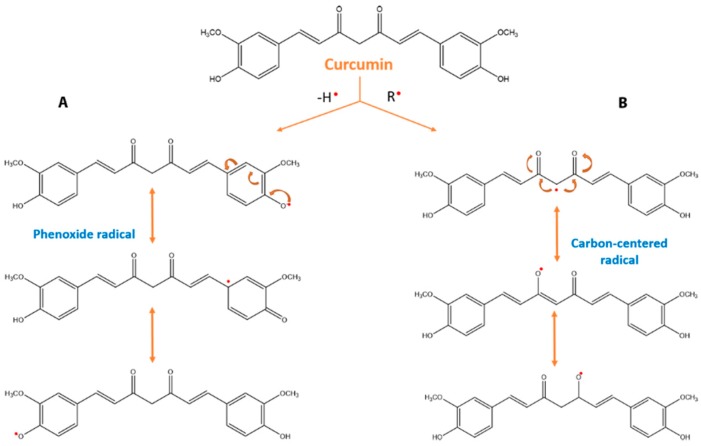
Antioxidant mechanism of curcumin. There are two mechanisms to form phenoxyl radicals. The first mechanism (**A**) begins by initial electron transfer to the free radical; thus, a radical cation is formed, which produces a phenoxyl radical by a proton loss. The second mechanism (**B**) is related to direct hydrogen abstraction. Based on the bond dissociation energies, many authors suggest that the most susceptible target for free radicals in curcumin is phenolic OH.

As previously mentioned, curcumin possesses a wide range of biological functions, including antioxidant activity. Curcumin contains a variety of functional groups, including phenolic rings that act as electron traps to prevent H_2_O_2_ production and scavenge superoxide radicals, the β-diketo group that is involved in metal–ligand complexation, and carbon–carbon double bonds (Figure 9) [56,58]; together, these provide the molecule with unique antioxidant properties.

Under acidic to neutral conditions (pH = 3−7), the keto conformation is the major form. On the other hand, under basic conditions (pH > 8), the enol conformation is predominant, which presents powerful free radical-scavenger properties [59].

Using erythrocyte sedimentation rate (ESR), the reduction of ferric iron in aqueous medium, and intracellular ROS/toxicity assays, Barzegar et al. [60] analyzed the antioxidant properties of curcumin. Their results demonstrated that curcumin confers protection on the cells against the mortal effects of cumene hydroperoxide.

In addition, Zbarsky et al. [58] applied, in the unilateral 6-hydroxydopamine (6-OHDA) rat model of PD, natural compounds, with phenolic groups in their structure to evaluate the neuroprotection level. These authors reported that, with the sub-chronic administration of curcumin, the loss of dopaminergic neurons in the *substantia nigra pars compacta* was reduced significantly.

The antioxidant protective effect of curcumin was also evaluated against hemin-induced neuronal death. In 2013, González-Reyes et al. [61] evaluated the neuroprotection of curcumin in the primary cultures of the cerebellar granule neurons of rats. They concluded that the use of curcumin as a pretreatment induces antioxidant protection against hemin-induced neuronal death.

The antioxidant effect of curcumin has been evaluated in other applications in addition to neuronal pathologies. For example, Haryuna et al. [62] investigated the antioxidant action of curcumin against oxidative stress caused by diabetes mellitus. These authors worked with cochlear fibroblasts in rat models of diabetes mellitus, and reported that curcumin confers antioxidant protection via the increased expression of superoxide dismutase. These results showed that curcumin presents a wide range of applications.

## 4. Limitation of Chemical Properties and New Proposals

There are two main limitations when curcumin is formulated for therapeutic purposes: limited solubility in water and low permeability [63]. In addition, the low permeability is related to chemical degradation, a high rate of biotransformation, especially glucuronidation and sulfation [22], and rapid systemic elimination, resulting in low curcumin absorption and poor bioavailability. Therefore, curcumin can be classified as a BCS Class IV molecule. In fact, the majority of oral curcumin is excreted in the feces—about 90%—and the rest in urine (6%) after 72 hours in rats [64]. Consequently, high doses of curcumin are necessary to produce detectable plasma concentrations. For example, in a human clinical trial, 3.6 g of curcumin via the oral route was found to produce a plasma curcumin level of 11.1 nmol/L after one hour of dosing on days one, two, eight, and 29 of the administration [65]. Due to these limitations, novel drug delivery systems have been proposed to increase the bioavailability of curcumin.

## 5. New Formulations of Curcumin

As previously mentioned, one of the reasons that curcumin has poor bioavailability is due to its limited solubility in water; therefore, one of the directions in the development of new formulations for this molecule (as for other lipophilic compounds) is to increase solubility, following strategies such as modification of the solid state, reduction of particle size, the creation of supersaturated solutions, or the encapsulation into nanoparticles. Some new formulations are briefly described later.

Amorphous solid dispersions (ASD) are an interesting strategy of modification of the solid state to improve the rate of dissolution of drugs, and thus their bioavailability. Solid dispersion involves the incorporation of water-insoluble compounds into a hydrophilic carrier matrix. In a study performed by Gangurde et al. [66], the authors demonstrated that curcumin formulated in an Eudragit E (Evonik Nutrition & Care GmbH, Essen, Germany) polymeric matrix dissolved more rapidly (20–45% release after 60 min) than curcumin alone (2–5% release after 120 min) at pH 1.2. Solubility also was increased: with curcumin alone, solubility was 0.02%, whereas curcumin containing Eudragit E (Evonik Nutrition & Care GmbH) exhibited solubility of 40.29% and 18.78% by the spray-drying and rotoevaporation techniques, respectively. In other studies, curcumin revealed an increase of >1000 times when it was formulated as ASD in hydroxypropyl methylcellulose matrixes [67].

Nanosuspension is a carrier-free nanoparticle system containing only pure drug crystals, and is sometimes accompanied by a stabilizer. Nanosuspension can greatly increase the saturation solubility as well as the dissolution velocity (by increasing the superficial area). Curcumin has been formulated as a nanosuspension with good results. For example, Wang et al. [68] found an increase in the oral bioavailability of curcumin of about three to four times after the administration of curcumin in nanosuspensions in rats. Similarly, Li et al. [69] found an increase in bioavailability after the intravenous administration of a nanosuspension of about 4.2-fold compared to a curcumin solution.

Self-microemulsifying drug delivery systems (SMEDDS) are isotropic mixtures of oil, hydrophilic surfactant, and co-solvents that rapidly form oil-in-water (o/w) microemulsion upon gentle agitation followed by dilution in an aqueous medium [70]. This class of supersaturated systems has been effective in increasing the solubility of drugs at the absorption site. Formulated curcumin in SMEDDS has been proposed by Wu et al. [71]. The formulation, which is composed of 20% ethanol, 60% Cremophor RH40 (BASF Personal Care and Nutrition GmbH, Monhein, Germany) and 20% isopropyl myristate, improved the relative oral bioavailability of SMEDDS compared with the curcumin suspension by about 1213%. Similar studies conducted by other researches can be found in the references [72,73,74].

**Figure 10:**
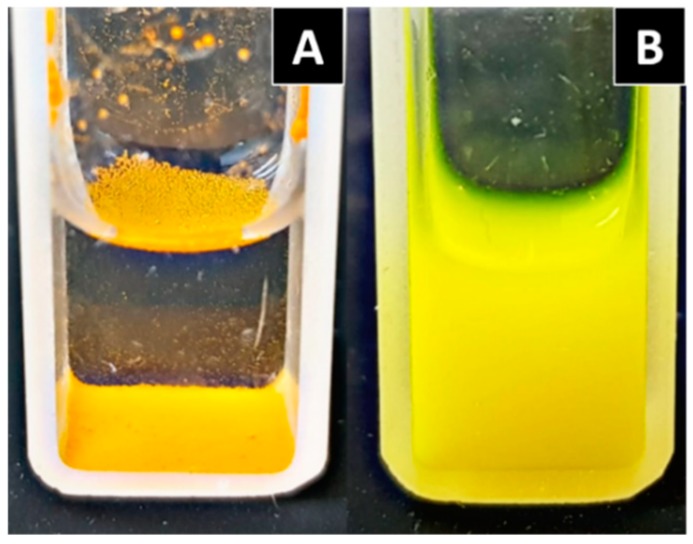
Curcumin solubility: (**A**) Curcumin showed poor solubility in aqueous medium; (**B**) the use of the nanoplatforms increased the drug solubility. Curcumin was entrapped in poly-ε-caprolactone nanoparticles stabilized by Pluronic F68 (Thermofisher, Whaltam, USA), with size of 170 nm and zeta potential of −7 mV.

In addition, the encapsulation of curcumin into nanoparticles not only enhances its bioavailability and solubility (Figure 10), it also increases its stability by protecting it from the influence of the outside environment.

Novel curcumin-nanoparticulated delivery approaches, including liposomes, polymeric nanoparticles, solid lipid nanoparticles, polymeric micelles, and others, are emerging as suitable and promising systems. This is because they can be designed and adapted to the desired size, chemical composition, surface charge, and surface functionalization, rendering them potent tools for the treatment of specific diseases such as neurodegenerative disorders and cancer [75,76]. In this review, a more detailed description on the development of formulations based on nanoparticles, specifically for neurodegenerative disorders are discussed in the following sections.

## 6. Formulations of Curcumin in Nanoparticles

Nanoparticles for medical purposes comprise a variety of drug transport systems; they traditionally have dimensions of fewer than 200 nm for brain applications, and are expandable up to 1000 nm, in concept. The small aggregation size confers a high degree of tissue penetration. Nanoparticles for medical and brain purposes are considered non-invasive systems. Although they are designed to flank and cross various tissues, their small size and composition guarantee, in the majority of cases, minimal invasion without risk. The main security provided comprises the chemical composition and the mechanisms of elimination and/or biodegradation. However, several research groups that have delved into this topic have always highlighted the need for a broad assessment in the area of nanotoxicology, because some materials, only by the change of size of aggregation from bulk to nanoparticles can increase their toxicity; contrariwise, other materials exhibit new and better properties.

Conventionally, nanoparticles for medical purposes arise from the combination of two master ideas: from Paul Ehrlich with the concept of magical bullets [77], and Richard Feynman with the notion of miniaturization [78]. However, the materialization of the investigation of new carriers for the transport of drugs to the brain with the use of nanoparticles dates from the first conceptions of Peter Speiser in 1969 [79]. Subsequently, J. Kreuter et al. [80] began the initial assessments on the coating of nanoparticles with polysorbate 80 to facilitate their passage into the CNS. These pioneering works set the standard for the development of polymeric nanoparticles. In parallel, R.H. Müller et al. [81] worked on the development of lipid nanoparticles that would later allow for wide applications in the same area.

The subsequent epochs were accompanied by pharmacokinetic and mechanistic studies at the cellular level with the intention of achieving greater degrees of vectorization. Therefore, some of the examples cited in the application of nanoparticles for the transport of curcumin to the CNS have employed as a strategy the coupling of ligands that permit targeting to the brain. Drug vectorization has advanced substantially, even to the achievement of reaching intranuclear levels in neurons. Some of the most commonly used systems for administering curcumin for the purpose of brain impairment are described below.

### 6.1. Polymeric Nanoparticles

**Figure 11:**
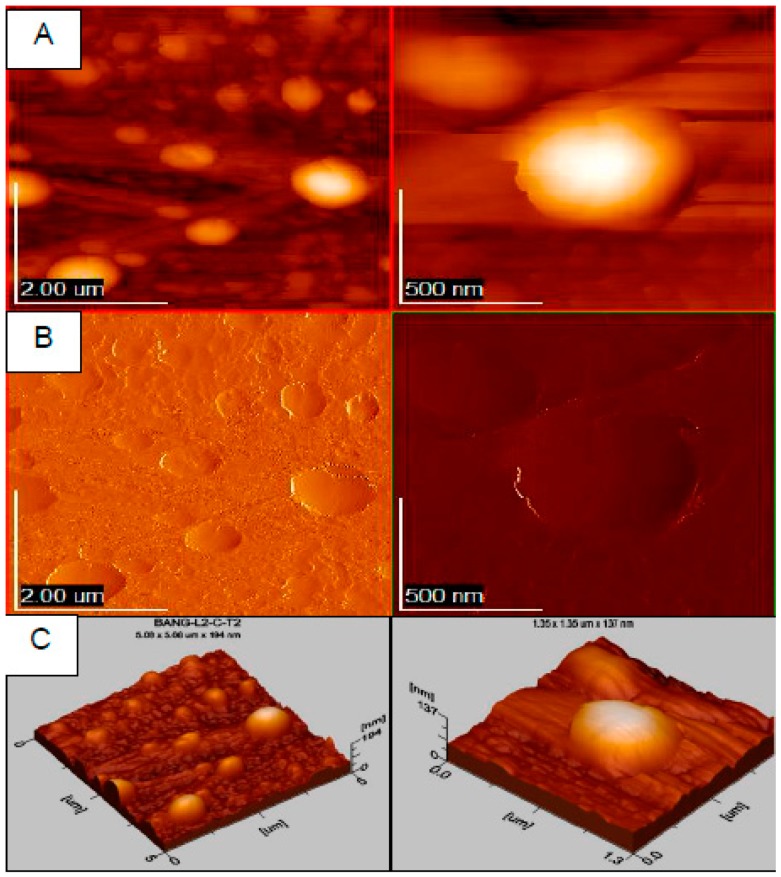
Atomic force microscopy (AFM) microscopy of curcumin poly-ε-caprolactone nanoparticles in two magnifications, left and right. Images in AFM (**A**), 2D (**B**) and 3D (**C**) mode.

Polymeric materials were the first to have been used to transport drugs to the brain, and a significant number of curcumin-carrying applications can be found in the literature. Some examples of traditional polymers include poly (butyl cyanoacrylate) (PBCA), poly (lactic-co-glycolic acid) (PLGA), and chitosan. In particular, the synthetic polymers PBCA and PLGA allow adequate control of the particle size, which results in highly reproducible production batches (Figure 11).

Subsequent steps have been directed toward the synthesis of new polymers to couple different ligands that can permit adequate vectorization. Usually, these systems allow suitable stability with a zeta potential of at least –15 mV with polyvinyl alcohol (PVA), while with systems prepared with chitosan, although they appear very promising by the simple and rapid methods of ionic gelation, one of the disadvantages is usually the control of molecular weight. In general, PLGA polymer nanoparticles have shown adequate biocompatibility at the cellular level, and in studies in more complex biological models, even at high amounts up to one mg/mL at a cell density of 1 × 10^4^. However, other studies have indicated that the degradation of PLGA could induce cellular damage by the generated acidic medium, although this is restricted to the amount of polymer that was administered [82].

### 6.2. Lipid Nanoparticles

Even when a broad demonstration of the biocompatibility of polymeric systems is available, doubt remains concerning their possible effects in prolonged administrations, due to the possible interactions within the nanometric range with biological systems or accumulation in some tissues. One of the main alternatives to the possible effects deriving from polymers are lipid nanoparticles. This term includes solid lipid nanoparticles and nanostructured lipid carriers. One of the great advantages of these systems is the use of lipids with high biocompatibility and biodegradation. The majority of lipid matrices are constituted of glyceride derivatives that are easily assimilated by our metabolism. We can imagine that several of these are also present in foods, and that they follow the same route of biotransformation. Therefore, this aspect is crucial in the choice of the type of nanoparticle matrix for the transport of curcumin, and it is reflected in a considerable percentage of the published information for brain diseases. The majority of production methods employ temperatures above the melting point of the lipids used. One aspect to be considered is the possible stability of lipid nanoparticles in aqueous dispersion and the exchange between polymorphic structures that can promote the expulsion of the drug. Interestingly, some lipids exhibit biological properties in addition to the nutritional aspect, and their applications are enhanced when handled in the nanometer state. Some of these demonstrate novel biological properties due to an increase in interaction with the biological environment, and to the high degree of distribution and penetration in tissues. If this were the case, the new properties that were exhibited can be complemented with the drugs encapsulated in the same system.

### 6.3. Liposomes

Liposomes are spherical vesicles with one or several layers of phospholipids with an internal aqueous compartment. They usually have a size of less than 100 nm. Due to the high percentage of phospholipids, they are also recognized as highly biocompatible and biodegradable systems. The difference with the previous systems, besides the composition, is the type of architecture. Lipid nanoparticles derive from a solid and compact matrix, and the liposomes do not; even there are some variants that confer flexibility to the structure. The development and evaluation of liposomes is evidenced by a long history in pharmaceutical technology. In fact, the first drug approved by the Food and Drug Administration (FDA) in the nano order was with liposomes, i.e., Doxil (Janssen, Titusville, FL, USA) for the treatment of certain types of cancer [83]. The long trajectory in liposome research also confers the establishment of well-implemented industrial processes that facilitate the subsequent development of similar formulations. This is somewhat different from the polymeric and lipid systems, in which there remain critical systems to be reinforced, especially when they involve high agitation speeds.

### 6.4. Cyclodextrins

Cyclodextrins are glucopyranose structures in the form of a glass with a hydrophobic interior and a hydrophilic exterior by the exposure of hydroxyl units [84]. Cyclodextrins can be used to transport drugs to different organs, and can also be derivatized to increase their vectorization. They have shown high biocompatibility for deriving from the starch. Unlike previous systems, cyclodextrins are prefabricated carriers for the direct incorporation of the cargo. In theory, competition is mentioned between the liquid medium inside the vessel and the new molecule that one wishes to enter. Therefore, it is necessary to know the constants of affinity and dissociation. As with a traditional glass, when transported to the table, the interior can spill. In the case of drugs, the cargo may also get in the way. The hydrophobic characteristic allows some lipid extraction at the BBB level, which could also facilitate passage into the brain region. Even in other pathologies, effects can be found with empty cyclodextrins due to the effect generated in lipophilic components.

### 6.5. Tools for Drug Targeting of Curcumin

The BBB is a highly sophisticated barrier that is designed to maintain the integrity and homeostasis of the brain. From a technological point of view, it is a great challenge to direct different carriers toward the brain region under the concept of “non-invasive”. Some strategies that have been employed include the surface coupling of the nanoparticles of ligands to specific receptors, the coupling of transporter substrates overexpressed in the BBB, and transport by adsorption through the interaction of opposite electrical charges (negative of the endothelial cell membrane and positive of the carrier). In other situations, various pathologies related with neuroinflammation produce a relative opening of the narrow junctions, therefore increasing the probability of the passage of carriers into the nanoparticle architecture. Intentional opening has not comprised a viable option because of the risk involved. Other strategies include furtive aggressive participation. The use of peptides, some of these from virus fragments or modified versions to ensure efficacy without toxicity, is a highly effective option. The transactivating-transduction peptide (TAT) has been a classic example of this type of mechanism.

It is noteworthy that the presence of surface agents substantially modifies the targeting capacity of the curcumin nanoparticles toward the CNS. However, a study in the literature mentions the formulation of PLGA nanoparticles with curcumin obtained by flash nanoprecipitation without a stabilizing agent. These authors demonstrated a 20-fold reduction in the administered dose compared with bulk curcumin (see Table 1). It is even more important to point out that the majority of the studies mentioned in Table 1, some of these that have been widely extended in biological elucidation mechanisms do not involve the application of highly sophisticated systems. In other brain pathologies, it has become a challenge to possess the most sophisticated. On the other hand, some formulations of curcumin in nanoparticles even include the combination of two active molecules, such as puerarin and dexanabinol. In addition, in the search for multifunctional systems, or theranostics, curcumin has been conjugated in magnetic nanoparticles for the detection of Aβ plaques in AD and in combination with gold–iron oxide nanocomposite systems for brain-cancer theranostics (see Table 1 for details).

## 7. Applications of Curcumin in Nanoparticles

### 7.1. Alzheimer’s Disease

Alzheimer’s disease is the most frequent neurodegenerative disorder worldwide; it is characterized by extracellular Aβ aggregation, intracellular neurofibrillary tangles, tau hyperphosphorylation, and progressive neuron loss. Its main clinical features are memory loss, behavioral changes, and cognitive impairment [126,127]. At present, the limited efficacy of the available therapies has encouraged the search for new treatments. In this regard, recent investigations undertaken in diverse experimental models have suggested that curcumin could be helpful for AD treatment. However, limitations in the bioavailability of curcumin have hindered its use and promoted the search for suitable vehicles to improve its pharmacological activity, such as nanoparticle formulations.

In recent years, the development of nanocarriers to transport curcumin to the brain in AD models has increased considerably; however, the efficacy, safety, and suitability of these formulations is still a main concern. Thus, in addition to the cellular uptake, the evaluation of cell viability has become pivotal to demonstrate the biocompatibility of these formulations.

It has been suggested that aluminum is involved in the etiology of AD; thus, in an early study, the neuroprotective effect of solid lipid nanoparticles loaded with curcumin (SLN-Cur) was proved in an AD mouse model induced by 100 mg/kg of aluminum chloride [91]. The results showed that treatment with SLN-Cur (50 mg/kg) produced a 73% recovery in acetylcholinesterase and a 97.46% recovery in membrane lipids with respect to the group treated with aluminum chloride alone. In addition, mice treated with SLN-Cur underwent less learning impairment and cognition loss, as measured by the Morris water maze test, which suggests the potential of SLN-Cur as an alternative therapeutic for AD.

Similarly, evidence for a reduction of neurogenesis in AD has been shown, which suggests that the induction of this neuronal process mediated by endogenous neuronal stem cells (NSC) would be a probable therapeutic target. In this regard, Tiwari et al. [105] explored the effect of PLGA nanoparticles loaded with curcumin (PLGA–NP–Cur) on neuronal differentiation and NSC proliferation. Through transmission electron microscopy (TEM) analysis, these authors showed that PLGA-NP-Cur had the ability to internalize into NSC derived from the hippocampus. Moreover, Alamar blue reduction and MTT assays suggested that PLGA-NP-Cur (0.001–50 µM) increased NSC proliferation and cell viability, with the highest effect at 0.5 µM. It is noteworthy that bulk curcumin enhanced NSC proliferation only at 0.5 µM, whereas PLGA-NP-Cur significantly increased NSC proliferation at much lower doses (0.001 µM, 0.01 µM, 0.1 µM, and 0.2 µM). Likewise, the results demonstrated that PLGA-NP-Cur reversed deficits in hippocampal neurogenesis and in learning and memory dysfunction in an AD rat model induced by the brain injection of Aß. Interestingly, a more pronounced effect was exhibited by PLGA-NP-Cur when compared with bulk curcumin. Collectively, the results suggested that PLGA-NP-Cur could offer an alternative for the treatment of AD and regenerative medicine.

On the other hand, diverse evidences suggest that oxidative stress participates in the pathophysiology of AD; thus, Djiokeng Paka et al. [128] evaluated the antioxidant and anti-inflammatory activities of curcumin-loaded PLGA nanoparticles (Cur–PLGA–NP) in SK-N-SH cells, which are human neuroblastoma-derived cells. Through assays of radical scavenging activity, the authors found that Cur-PLGA-NP exhibited 1.5-fold and 2.2-fold greater antioxidant activity against peroxyl radical than curcumin alone and empty NP, respectively. They also showed that Cur-PLGA-NP prevented Tau phosphorylation and Akt activity, which have been shown as altered in AD brains. Therefore, the authors suggested that Cur-PLGA-NP could be potentially useful for treating AD.

In another study, Meng et al. [94] developed a low-density lipoprotein-mimic nanocarrier attached to lactoferrin encapsulated in curcumin (Lf–mNLC–Cur) and evaluated its effect on the progression of disease in a rat model of AD. The rat model was generated by means of an intraperitoneal injection of D-gal (0.3 mL/100 g/d for six weeks) and a bilateral injection of Aβ_1-42_ in the dorsal hippocampus (one mg/mL; 5 µL). Through fluorescence images, it was shown that Lf-mNLC-Cur had the ability to cross the BBB, to penetrate into the brain, and to release curcumin. In order to evaluate the therapeutic effect of the formulation, histological preparations to examine the state of the nerve cells in the hippocampal region after the administration of Lf-mNLC-Cur and measurements of malondialdehyde (MDA) in blood were performed. Hematoxylin–eosin staining revealed a lower damage in the treatment with Lf-mNLC-Cur, and the content of MDA was reduced with respect to the control, which suggested that the formulation was effective for decreasing the oxidative stress associated with the progression of AD [94].

Similarly, Barbara et al. [129] designed and engineered a curcumin-encapsulated PLGA nanoparticle bound to peptide g7 (Cur-NP-g7) to promote BBB crossing. The authors demonstrated that Cur-NP-g7 can internalize hippocampal neurons, and that concentrations to around 200 µM were not toxic. Furthermore, the authors determined the effect of Cur-NP-g7 on Aβ aggregation, employing an in vitro AD model generated in the primary hippocampal culture derived from the rat brain. The results revealed an important decrease of Aβ; thus, they concluded that their formulation represent a promising tool for the treatment of AD.

On the other hand, a recent study demonstrated the anti-inflammatory effects of curcumin-loaded spherical polymeric nanoparticles (Cur-SPN). In that study, it was demonstrated that Cur-SPN (10 µM) reduced cytokines IL-1ß, IL-6, and TNF-α expression in macrophages stimulated by Aβ fibrils (2 µM). Likewise, cell viability assays revealed that Cur-SPN did not induce apoptosis, and no evidence of toxicity was detected. Based on their findings, the authors claimed that this approach could be helpful for the amelioration of inflammation observed in AD [94].

In a study undertaken by Huang et al. [130], PLGA nanoparticles were designed conjugated with the S1 peptide (an inhibitor of Aβ generation), brain-targeting calreticulin (CRT) (a peptide that binds to the transferrin receptor), and curcumin (as a therapeutic substance to tackle the disorder). The results revealed that the nanocarrier (S1-CRT-NP+Cur) was taken up into a cellular model of the BBB (brain microvascular bEnd.3 cells), suggesting that it can permeate across the BBB. In support of this hypothesis, S1-CRT-NP+Cur was distributed in mouse brain after intravenous administration, as demonstrated by in vivo bioluminescence imaging system and post-mortem studies. The therapeutic effect of S1-CRT-NP+Cur was explored by means of a Y-maze and a new object recognition test, the determination of astrogliosis and microgliosis, and the measurement of cytokines, superoxide dismutase (SOD), and ROS levels in a transgenic AD mouse model (APP/PS1dE9). Interestingly, S1-CRT-NP+Cur attenuated cognitive deficits, reduced astrogliosis and microgliosis, increased the number of synapses, decreased inflammatory cytokines, enhanced SOD levels, and reduced ROS levels. These findings highlight S1-CRT-NP+Cur as a promising approach for future use in AD treatment.

Finally, in a recent report, PLGA–PEG nanoparticles attached to the B6 peptide with curcumin encapsulated (PLGA/PEG-B6-Cur) were designed [108]. PLGA–PEG was employed to enhance the bioavailability, and the B6 peptide was employed to permit BBB crossing. In order to explore their potential usefulness in AD treatment, PLGA/PEG-B6-Cur were proven in HT22 cells and an AD rodent model (APP/PS1 transgenic mice). Cytotoxicity studies in HT22 cells demonstrated that PLGA/PEG-B6-Cur did not affect cell viability up to 500 µg/mL. To evaluate the effect of PLGA/PEG-B6-Cur on memory capability and spatial learning, the Morris water maze test was performed in animals intraperitoneally injected with the nanocarrier. The experimental results revealed that PLGA/PEG-B6-Cur significantly improved cognitive performance, which correlated with a decrease in tau phosphorylation and Aβ production in the hippocampus. Altogether, these findings suggest that PLGA/PEG-B6-Cur may represent a promising alternative for treating AD.

### 7.2. Parkinson’s Disease

Parkinson’s disease is the second most frequent neurodegenerative disorder and is characterized by abnormalities in the control of voluntary movements. The pathological hallmarks of PD include neuronal cell death, oxidative stress, mitochondrial dysfunction, and the accumulation of α-synuclein. The gold standard for the treatment of PD is l-Dopa; however, its chronic employment leads to a severe collateral problem known as dyskinesia. Therefore, the lack of a permanent cure has triggered the search for novel treatments. In this respect, curcumin has shown good effects in different experimental models; thus, it could be a key molecule for developing new strategies to reach a definitive treatment.

In this regard, in a first approach, alginate–curcumin nanoparticles (Alg-NP-Cur) were developed, and their probable therapeutic actions were evaluated in a PD Drosophila model. Alg-NP-Cur exhibited antioxidant power by the reduction of the lipid peroxidation in the PD Drosophila brain after a diet supplemented with the nanocarrier for 24 days. The effective prevention of the progression of the Parkinsonian symptoms in the PD flies was evidenced by the inhibition of the loss of climbing ability in flies with respect to PD flies without exposure to Alg-NP-Cur. Therefore, this report provided a rationale for employing curcumin-encapsulated nanoparticles in preclinical and clinical studies [131].

In this respect, in another study, Kundu et al. [88] designed glyceryl monooleate nanoparticles loaded with piperine and curcumin (GMO-NP-Pip/Cur). The anti-aggregate and anti-fibrillar effects of GMO-NP-Pip/Cur on α-synuclein oligomers were evaluated employing atomic force microscopy (AFM). The results showed that GMO-NP-Pip/Cur produced a pronounced inhibition of α-synuclein aggregation. Similarly, GMO-NP-Pip/Cur were able to ameliorate motor dysfunction when co-administered with rotenone in a PD mouse model. Interestingly, GMO-NP-Pip/Cur exhibited anti-apoptotic and antioxidant activities with no evidence of cytotoxicity. Therefore, GMO-NP-Pip/Cur could be an interesting approach for the treatment of PD.

In another study, Bollimpelli et al. [132] elaborated lactoferrin nanoparticles by sol-oil chemistry, which were loaded with curcumin (Lf-NP-Cur). The potential neuroprotective usefulness of this formulation was evaluated against toxicity induced by rotenone in SK-N-SH cells, employing microscopy, a lactate dehydrogenase (LDH) release assay, and an MTT assay. The findings suggested that Lf-NP-Cur protect the cells from the toxicity of rotenone, more than curcumin alone; thus, these may be a suitable drug-delivery strategy against PD.

Finally, nanoparticles with Polysorbate 80 are widely used to improve the BBB permeability of the nanoparticles; thus, Zhang et al. [133] developed curcumin-loaded polysorbate 80-modified cerasomes (PS80-NP-Cur). The formulation was able to modify the release time of curcumin monitored in C57BL/6 mice, producing a prolonged circulation time in the blood. Furthermore, PS80-NP-Cur produced a remarkable reduction of PD symptoms in a PD mouse model induced by 1-methyl-4-phenyl-1,2,3,6-tetrahydropyridine (MPTP), when these were administered concomitantly with ultrasound-targeted microbubble destruction (UTMD). Despite these promising results, the utilization of techniques such as UTMD should be evaluated to a greater extent, because its employment for prolonged times could lead to severe undesirable effects.

### 7.3. Huntington’s Disease

Huntington’s disease is a genetic neurodegenerative disorder whose clinical manifestations include involuntary movements and anxiety. To date, there is no specific medication to treat the disease; however, since mitochondrial impairment appears to contribute to neuronal death in HD, approaches to decrease mitochondrial dysfunction could represent a therapeutic alternative. In this regard, it has been reported that dietary curcumin possesses beneficial effects in HD. Therefore, Sandhir et al. [87] fabricated solid lipid nanoparticles loaded with curcumin (SLNP-Cur) and explored their neuroprotective effectiveness in a HD rat model induced by 3-nitropropionic acid (3-NP). The administration of SLNP-Cur was able to restore SOD activity and glutathione levels. Furthermore, SLNP-Cur decreased ROS, protein carbonyls, and lipid peroxidation. In addition, the formulation reduced the motor impairment induced by the treatment with 3-NP. Therefore, these results indicate that SLNP-Cur could be helpful for the treatment of HD.

## 8. Conclusions

Curcumin is an example of an ancestral phytochemical whose health benefits have been confirmed and new applications have been discovered with a high impact for incurable diseases. The study of curcumin is one of the few cases of broad applications demonstrated under methodological principles and in a reproducible manner. Recently, the greatest impacts of this molecule are due to the novel technological proposals for its biological administration. In particular, the use of nanoparticles has made it possible to demonstrate benefits at the brain level, which can even revolutionize medicine. However, as with any chemical substance, it is convenient to emphasize toxicity issues in order to ensure the safety of all clinical trials. In addition to the clear results in brain models, the increase in benefits can be addressed with an improvement in the functioning of nanotechnological carriers.

## Figures and Tables

**Table 1:** Examples of nanoparticle formulation for transport of curcumin to the brain.

Carrier	Composition	Ligand/Stabilizer	Size, PI and Ψ (mV)	% of EE and DL	Model of Evaluation	Reference
Solid lipid nanoparticles	Polyoxyethylene stearate, stearic acid	Lecithin	60, Ψ = −21.7	DL = 21.61	Major depression (in vitro and in vivo models)	[85]
	Compritol 888 ATO (Gattefossé, Saint-Priest, France)	Tween 80, soya lecithin	136	81.9, 92.3	Cerebral ischemic injury (in vivo model)	[86]
	Stearic acid	Lecithin, taurocholate	148	EE = 93.2	Huntington’s disease (in vivo model)	[87]
	Glyceryl monooleate	Pluronic F-68, vitamin E TPGS	93, Ψ = −30.9	EE = 65	Rotenone-induced mouse model of Parkinson’s disease (in vitro and in vivo models)	[88]
	Palmitic acid, cholesterol	N-trimethyl Chitosan vitamin E TPGS	412, 0.26, Ψ = 35.7	93, 4	Biodistribution (in vitro and in vivo models)	[89]
	Compritol 888 ATO	Tween 80, soya lecithin	136	81.9, 92.3	Aluminum-induced behavioral (in vivo model)	[90]
Solid lipid nanoparticles (SLNs) and nanostructured lipid carriers (NLCs)	Cetyl palmitate (SLN), cetyl palmitate + oleic acid (NLC)	Tween 80	204.7, 0.194 and 117.36, 0.188	SLN = 83.98, 4.54 NLC = 82.60, 4.67	Pharmacokinetic (in vivo model)	[91]
Nanostructured lipid carriers	Precirol, capmul MCM	Tween 80, soya lecithin	146, 0.18, Ψ = −21.4	EE = 90.86	Astrocytoma-glioblastoma (in vitro and in vivo models)	[92]
	Glyceryl monostearate, soy lecithin, medium chain triglycerides	Poloxamer 188	129, 0.25 Ψ = −27.8	95.9, 4.21	Pharmacokinetic and biodistribution (in vivo model)	[93]
	Phosphatidyl choline, cholesterol oleate, glycerol trioleate	Lactoferrin	103.8, PI = 0.15, Ψ = −5.80	96.51, 2.60	Alzheimer´s disease (in vitro and in vivo models)	[94]
	PC, cholesterol oleate, glycerol trioleate	Polysorbate 80	90.5, 0.14, Ψ = −20.3	EE = 94.39, DL = 3.29	Biodistribution (in vitro and in vivo models)	[95]
Polymeric nanoparticles	PLGA	Lipid monolayer	193.4, PI=0.115, Ψ = −43.8	13.23, 2.31	Inflammation model (*in vitro* model)	[96]
	Poly(butyl) cyanoacrylate	Apolipoprotein E3	197, 0.18, Ψ = −22.44	-	Beta amyloid induced cytotoxicity in neuroblastoma cells (*in vitro* model)	[97]
	Poly(butyl) cyanoacrylate	Apolipoprotein E3	197, 0.18, Ψ = −22.44	EE = 77.85	Anticancer activity in neuroblastoma cells (*in vitro* model)	[98]
	PLGA	-	100	94.7, 47.3	Bioavailability in the CNS (*in vivo* model)	[99]
	PLGA	PVA	163, 0.053, Ψ = −12.5	EE = 46.9	Pharmacokinetic (*in vivo* model)	[100]
	PLGA	-	-	-	Opioid tolerance and dependence (*in vivo* model)	[101]
	Chitosan	Tween 80	10, Ψ = −16.8	-	Arsenic toxicity (*in vivo* model)	[102]
	Chitosan-alginate	-	50	-	Epilepsy (*in vivo* model)	[103]
	Chitosan	Bovine serum albumin	143.5, 0.021, Ψ = −10.8	EE = 95.4	Phagocytosis of the Aβ peptide (*in vitro* model)	[104]
	PLGA	PVA	200, Ψ = −19	EE = 77	Neurogenesis (*in vitro* and *in vivo* models)	[105]
	PLGA	PVA	153, 0.15	90, 9.5	Pain (*in vivo* model)	[106]
	PLGA	Tet-1	150-200, Ψ = −30 to −20	-	Amyloid aggregates (*in vitro* model)	[107]
	PLGA	PEG-B6 peptide	150, Ψ = 3.8	DL = 15.6	Alzheimer transgenic mice (*in vitro* and *in vivo* models)	[108]
	PLGA	PEG, cyclic hexapeptide	97.3, 0.16	EE = 80.5	Glioma tumor cells (*in vitro* and *in vivo* models)	[109]
	PLGA	PEG, transferrin receptor-binding peptide T7	130, Ψ = −15.9	EE = 18	Brain tumor (*in vitro* and *in vivo* models)	[110]
	PLGA	1,2-distearoyl-glycerol-3-phospho-ethanolamine- N-[methoxy (polyethylene glycol)-2000	169, 0.22	EE = 35	Glioblastoma (*in vitro* and *in vivo* models)	[111]
	PLGA	PVA	220, Ψ = −20.6	81.7, 16.3	Subarachnoid hemorrhage-induced BBB disruption (*in vivo* model)	[112]
	PLA–PEG	PVP	55, 0.09, Ψ = −0.29	EE = 99	Alzheimer’s Disease Tg2576 Mice (*in vitro* and *in vivo* models)	[113]
	Hyaluronic acid/chitosan	-	207, Ψ = 25.3	89.9, 6.5	Glioma cells (*in vitro* model)	[114]
Polymeric micelle	Oleoyl chloride, polyethylene glycol 400	-	142, 0.4, Ψ = −7	EE = 87	Glioblastoma cells (*in vitro* model)	[115]
Nanoemulsion	Labrafac Lipophile WL 1349, Solutol HS 15, Transcutol HP	Tween 80, Tween 20	67, 0.137, Ψ = −37	-	Malignant glioma cells (*in vitro* model)	[116]
	Labrafac Lipophile	Cremophor RH40	114, 0.25, Ψ = −21.8	-	Biodistribution (*ex vivo* and *in vivo* models)	[117]
	Castor oil	Soybean lecithin, PEG 660-stereate	20.7, 0.19, Ψ = −9.7	EE ≥ 99	Permeation in Franz cells (*ex vivo* model)	[118]
Metallic nanoparticles	Au	PEG	-	-	Lipopolysaccharide-induced inflammation (*in vitro* and *in vivo* models)	[119]
Magnetic nanoparticles	Iron (II) sulfate heptahydrate	PEG-PLA	94, 0.14, Ψ = −0.01	EE = 99	Detection of amyloid plaques in Alzheimer’s (*in vitro* and *in vivo* models)	[120]
	Gold-iron oxide	Glutathione	40, 0.185, Ψ = −16	EE = 70, 0.7	Brain cancer (*in vitro* model)	[121]
	Fe3O4	-	185, Ψ = −37.5,	EE = 75	Schizophrenic rats (*in vivo* model)	[122]
	Iron oxide. SPIO nanoparticles, 10,12-pentacosadiynoic acid	PVA, Lactoferrin	100	EE = 90.3	Orthotopic Brain Tumor-Bearing Rat (*in vitro* and *in vivo* models)	[123]
Liposomes	1,2-dipalmitoyl-sn-glycerol-3-phosphatidylcholine, cholesterol	-	207, 0.25, Ψ = −10.5	-	Amyloid peptide plaques (*in vivo* model)	[124]
β-cyclodextrin (BCD), nanoliposome (NL)	β-cyclodextrin, phosphatidylcholine:cholesterol (5:1)	Tween 80 for liposome	133.49, −31.76 and 121.81, −7.91	BCD = 76.6, 19.73 NL = 88.2, 4.13	Dimethylhydrazine induced poison (*in vivo* model)	[125]

PVA: polyvinyl alcohol, PLGA: poly (lactic-co-glycolic acid), PVP: polyvinylpyrrolidone, PI: polidispersity index, PEG: polyethylene glycol, EE: entrapment efficiency, DL: drug load, D-α-tocopheryl polyethylene glycol 1000 Succinate: vitamin E TPGS, CNS: central nervous system, BBB: blood–brain barrier.

## Abbreviations

AD	Alzheimer disease
AP-1	activating protein-1
APP	amyloid-β precursor protein
AR	androgen receptor
Arh-R	aryl hydrocarbon receptor
AS	α-synuclein
ASD	Amorphous solid dispersions
Aβ	amyloid-β
BACE1	β-secretase enzyme
BBB	blood–brain barrier
BCS	Biopharmaceutics Classification System
cAK	autophosphorylation-activated protein kinase
CBP	CREB-binding protein
CDPK	Ca^2+^-dependent protein kinase cellular src kinase
CNS	central nervous system
COX-2	cyclooxygenase-2
cPK	protamine kinase
CTGF	connective tissue growth factor
DFF40	DNA fragmentation factor; 40-kd subunit
DL	drug loading
DR-5	death receptor-5
EE	entrapment efficiency
EGF	epidermal growth Factor
EGF-R	EGF receptor
EGFRK	EGF receptor-kinase
Egr-1	early growth response gene-1
ELAM-1	endothelial leukocyte adhesion molecule-1; Bcl-2, B-cell lymphoma protein 2
EPC-R	endothelial protein C-receptor
EpRE	electrophile
ERK	extracellular receptor kinase
ER-α	estrogen receptor-α
FAK	focal adhesion kinase
Fas-R	Fas receptor
FDA	Food and Drug Administration
FGF	fibroblast growth factor
FPTase	farnesyl protein transferase
Gcl	Glutamate–cysteine ligase
GM-CSF	granulocyte-macrophage colony-stimulating factor
GST	glutathione-S-transferase
H2-R	histamine (2)-receptor
HGF	hepatocyte growth factor
HO	heme oxygenase
HSP-70	heat shock protein 70
IAP	inhibitory apoptosis protein
IARK	IL-1 receptor-associated kinase
ICAM-1	intracellular adhesion molecule-1
IFNγ	interferon gamma
IKK	IκB kinase
IL	interleukin
IL-8-R	interleukin-8-receptor
iNOS	inducible nitric oxide synthase
Inos	matrix inducible nitric oxide synthase
InsP3-R	inositol 1,4,5-triphosphate receptor
IR	Fourier transform infrared spectroscopy
IR	integrin receptor
JAK	janus kinase
JNK	c-jun N-terminal kinase
LDL-R	low-density lipoprotein-receptor
LOX	lipoxygenase
LPS	lipopolysaccharide
MAPK	mitogen-activated protein kinase
MCP	monocyte chemoattractant protein
MDR	multidrug resistance
MIF	migration inhibition protein
MIP	macrophage inflammatory protein
MMP	matrix metalloproteinase
Mv	Millivolts
NAT	arylamine N-acetyltransferases
NF-κB	nuclear factor-κB
NGF	nerve growth factor
NMR	nuclear magnetic resonance
Nrf-2	nuclear factor erythroid 2-related factor
PBCA	poly (butyl cyanoacrylate)
PDGF	platelet-derived growth factor
PhK	phosphorylase kinase
PKA	protein kinase A
PKB	protein kinase B
PKC	protein kinase C
PLGA	poly lactic-co-glycolic acid
pp60c-src	non-receptor protein tyrosine kinase c-Src
PPARγ	peroxisome proliferator-activated receptor-γ
PrP	prion protein
PrP-Res	prion protein resistance
PVA	polyvinyl alcohol
SHP-2	Src homology 2 domain containing tyrosine phosphatase 2
SMEDDS	Self-microemulsifying drug delivery systems
STAT	signal transducers and activators of transcription
TAT	transactivating–transduction peptide
TGF-β1	transforming growth factor-β1
TIMP	tissue inhibitor of metalloproteinase-3
TK	protein tyrosine kinase
TNF	tumoral necrosis factor
TNF-α	tumor necrosis factor-α
uPA	urokinase-type plasminogen activator
VCAM-1	vascular cell adhesion molecule-1
VEGF	vascular endothelial growth factor
Ψ	zeta potential
